# Does smallpox vaccination modify HIV disease progression among ART-naive people living with HIV in Africa?

**DOI:** 10.1017/S0950268817002795

**Published:** 2017-12-13

**Authors:** A. DIOUF, H. TROTTIER, T. J. YOUBONG, N. F. NGOM-GUÉYE, O. NDIAYE, A. SECK, D. SARR, S. DIOP, M. SEYDI, S. MBOUP, V. K. NGUYEN, A. JAYE

**Affiliations:** 1 Department of Infectious Diseases/Regional Research and Training Center on HIV and Associated Diseases, Fann's University Hospital Center, Dakar, Senegal; 2 School of Public Health, Department of Social and Preventive Medicine, Université de Montréal, Montreal, Canada; 3 Sainte-Justine University Hospital Research Center, 3175 Côte Sainte-Catherine, Montreal, Canada; 4 Ambolatory Treatment Center, Dakar, Senegal; 5 Pasteur Institute, Dakar, Senegal; 6 Health Promotion Center, Sida Service, Dakar, Senegal; 7 National Blood Transfusion Center, Dakar, Sénégal; 8 IRESSEF: Institut de Recherche en Santé, de Surveillance Épidémiologique et de Formation, Dakar, Sénégal; 9 Research Center of the Montreal University Hospital Center (CRCHUM), Montreal, Canada; 10 Medical Research Council Unit The Gambia, Banjul, Gambia

**Keywords:** Africa, ART-naïve, immune activation, smallpox vaccination

## Abstract

We examined the association between a history of smallpox vaccination and immune activation (IA) in a population of antiretroviral therapy-naïve people living with HIV (PLHIV). A cross-sectional study was conducted in Senegal from July 2015 to March 2017. Smallpox vaccination was ascertained by the presence of smallpox vaccine scar and IA by the plasma level of *β*-2-microglobulin (*β*2m). The association was analysed using logistic regression and linear regression models. The study population comprised 101 PLHIV born before 1980 with a median age of 47 years (interquartile range (IQR) = 42–55); 57·4% were women. Smallpox vaccine scar was present in 65·3% and the median *β*2m level was 2·59 mg/l (IQR = 2·06–3·86). After adjustment, the presence of smallpox vaccine scar was not associated with a *β*2m level ⩾2·59 mg/l (adjusted odds ratio 0·94; 95% confidence interval 0·32–2·77). This result was confirmed by the linear regression model. Our study does not find any association between the presence of smallpox vaccine scar and the *β*2m level and does not support any association between a previous smallpox vaccination and HIV disease progression. In this study, IA is not a significant determinant of the reported non-targeted effect of smallpox vaccination in PLHIV.

## INTRODUCTION

Smallpox was declared eradicated in 1980 by the World Health Organization (WHO). The threat of bioterrorism and developments in synthetic biology have fed concerns about the risk that smallpox might return according to the Independent Advisory Group on Public Health Implications of Synthetic Biology Technology Related to Smallpox [1]. Hence in 2016, the Advisory Committee on Variola Virus Research (ACVVR) recommended the maintenance of stocks of smallpox viruses, mainly for research purposes [2]. New smallpox vaccines have been developed and some are in different phases in clinical trials. Higher efficacy and safety profiles than vaccinia virus (VACV) have been reported, suggesting that newer vaccines could be administered to immunosuppressed persons including people living with HIV (PLHIV) [2, 3]. In this context, it seems important to study the epidemiological relationship between smallpox vaccination and HIV infection.

A link between smallpox vaccination and HIV infection has been suggested since the emergence of HIV epidemic. *In vitro* experiments have shown that a previous smallpox vaccination could provide a certain protection against HIV infection or disease progression [4, 5]. Previous studies conducted in African populations have also reported a positive effect on immune systems and on mortality [6, 7].

Recent data on the pathogenesis of HIV argue that chronic immune activation (IA) is the main factor driving the progression of HIV infection [8]. IA can be influenced by several factors, including co-infections and the frequency of sexual exposure to HIV. High levels of IA are reported in sub-Saharan Africa, where the majority of PLHIV are found. IA also depends on non-specific immunity and it has recently been shown that immunity due to the non-specific effect of a pathogen persists in humans and can confer strong and innate non-specific protection [9]. It has been suggested that smallpox vaccination could provide protection even in PLHIV. Moreover, some studies have shown that cellular and humoral immunity of VACV could persist for decades [10–12].

We hypothesised that PLHIV who had previously received smallpox vaccination would have a lower level of IA and a slower disease progression than those who had not.

Sub-Saharan Africa was the last WHO geographical region to eradicate smallpox [13]. In Senegal, smallpox vaccine was administered until 1980, when smallpox was declared eradicated. Senegal is a setting where IA levels are high and where one of the populations with the most recent smallpox vaccination is present. This offers the opportunity to evaluate the epidemiological relationship between a previous smallpox vaccination and HIV disease progression using the level of IA as a marker.

## METHODS

### Study design and population

We conducted a cross-sectional study between July 2015 and March 2017 on a population of antiretroviral therapy (ART)-naïve PLHIV at the « Service des Maladies Infectieuses et Tropicales/Centre Régional de Recherche et de Formation à la Prise en Charge du VIH et Maladies Associées (SMIT/CRCF) », Dakar, Senegal. This is a West African country with a concentrated HIV epidemic: HIV prevalence is low (0·7%) in the general population, but high in key populations such as people who inject drugs, men who have sex with men and female sex workers [14–17].

A study population of 101 ART-naïve PLHIV was recruited from the principal HIV treatment sites in the country: the SMIT/CRCF and the « Centre de Traitement Ambulatoire (CTA) » which are reference centres for the care of PLHIV, the « Centre de Promotion de la Santé (CPS) » which is a communal health centre and the « Centre National de Transfusion Sanguine (CNTS) » which is the national blood transfusion centre where HIV screening was systematically performed for every donor. PLHIV from these sites were included in our study if they: (1) were born before 1980, (2) were ART-naïve, (3) were not hospitalised at the time of the study and (4) signed the inform consent form.

The study protocol was approved by the institutional ethical and research review boards of the participating institutions in Senegal: (*Comité national d’éthique pour la recherche en santé* (CNERS) of the Ministry of Health) and in Canada (Research Ethical Board of Sainte-Justine University Hospital, Montreal).

### Data collection

Clinical data were collected during the medical visit and results of laboratory tests were obtained from patient's medical charts. These data were reported on a case report form designed for the study.

### History of smallpox vaccination

The exposure variable was the history of smallpox vaccination. This was ascertained if smallpox vaccine scar was visible on medical visits. The smallpox vaccine scar presents specific characteristics: it is broken, with a smooth central area, a rough peripheral rim and lines from the centre to the periphery. This differentiates it from the Bacillus of Calmette and Guerin (BCG) vaccine scar. The BCG vaccine is a viable avirulent attenuated strain of *Mycobacterium tuberculosis* which confers protection against certain forms of tuberculosis. The BCG vaccine scar is located on the upper left arm with a raised centre. Although both scars can be round or oval, smallpox scar is usually bigger with a diameter >10 mm, while BCG scar diameter is <10 mm. Two clinicians independently assessed whether or not the smallpox vaccine scar was present, and only concordant cases (agreement on presence or absence) were retained. The number of smallpox vaccine scars was specified as well as the diameter of the biggest one.

### *β*-2-Microglobulin measurement

The outcome variable was the *β*-2-microglobulin (*β*2m) level. Plasma levels of *β*2m were measured using the integrated automated Abbott Architect ci4100 system (Abbott Laboratories, Wiesbaden, Germany) in accordance with the instructions of the manufacturer; using Quantia *β*2m reagents under calibrated conditions. Thawed plasma samples that were collected in EDTA tubes were used for a single time point assessment. The results are expressed in mg/l of *β*2m based on the WHO International Standard [18].

### Other covariates

The other variables studied were socio-demographic characteristics (age, sex, marital status, occupation, education level), clinical data (body mass index (BMI), presence of BCG vaccine scar), variables related to HIV infection (HIV serotype, WHO clinical stage, CD4 cell count, CD4/CD8 ratio, plasma viral load (VL)) and presence of comorbidities (diabetes, hypertension, cardiovascular disease, chronic kidney disease, stroke, viral hepatitis B or C).

### Data analysis

The different characteristics of the study population were described for each study group (*presence of smallpox vaccine scar, absence of smallpox vaccine scar*). Comparisons of categorical variables were done using *χ*^2^ test. Continuous variables were compared between two groups by *t* test or Mann–Whitney/Wilcoxon test. The comparisons of continuous variables between three groups were done using analysis of variance or Kruskal–Wallis test.

The association between study group and *β*2m level was analysed by a logistic regression model with different cut-off points (median = 2·59 mg/l and fourth quintile = 4·73 mg/l). We estimated crude and adjusted measures of association (odds ratios (ORs)) with 95% confidence intervals (CIs). Confounding was controlled using a 10% change in estimate method (variables that change the estimate by ⩾|10%| were included in the model) among the following potential confounders: age (35–45/46–55/56–66), sex (male *vs* female), marital status (single, married, divorced, widower), education level (absence/elementary/high school/university), occupation (public or private sector executive, other public or private sector employee, informal worker, no current occupation), BMI (<18·5/18·5–24·9/⩾25), presence of BCG vaccine scar, HIV serotype (HIV-1, HIV-2, HIV-1 + HIV-2 dual infection), WHO clinical stage (stage 1 or 2, stage 3 or 4), estimated glomerular filtration rate (eGFR) (continuous), haemoglobin level (<10, ⩾10), plasma VL (log copies/mL, continuous) and presence of comorbidities (yes, no). Age, sex and presence of BCG vaccine scar were tested as effect modifiers. We also performed a linear regression model with *β*2m level (continuous) as outcome variable using the same methodology.

Statistical analyses were performed using the version 14 of Stata.

## RESULTS

### Study population

Between July 2015 and March 2017, we recruited 101 participants: 57·4% were women (95% CI 47·4–66·8). Median age, BMI, CD4 cell count and CD4/CD8 ratio were 47 years (interquartile range (IQR) = 42–55), 22·1 kg/m^2^ (IQR = 19·1–27·3), 411 cells/*μ*l (IQR = 149–580) and 0·43 (0·18–0·95), respectively. The majority of this population (50·5%) was comprised of informal workers, while 33·7% had no current occupation. A total of 29 participants (29·3%; 95% CI 21·1–39·2) had CD4 cell count <200 and 77 (77·8%; 95% CI 68·4–85·0) had a CD4/CD8 ratio <1. The plasma VL was >10 000 copies\mL for 76·9% of the study population; 95% CI 66·0–85·1. The predominant serotype was HIV-1: 74·2% (95% CI 64·7–81·9) with 21·8% of HIV-2 (95% CI 14·7–31·0) and 4·0% of HIV-dual infection (95% CI 1·5–10·2).

The immunisation record was available for only one participant but did not concern the period of interest (before 1980). Only one participant was able to confirm that he had received the smallpox vaccine. A proportion of 65·3% (95% CI 55·4–74·1) of the study population presented with a smallpox vaccine scar. Among them, the proportions of those with one, two or three scars were 74·2% (95% CI 62·1–83·5), 18·2% (95% CI 10·5–29·7) and 7·6% (95% CI 3·1–17·3), respectively. The average diameter of the largest scar was 17·3 mm (standard deviation (s.d.) = 7·4).

A BCG vaccine scar was found in 59·4% of the study population (95% CI 49·4–68·7).

The average *β*2m level was 3·26 mg/l (1·84) and the median level was 2·59 mg/l; IQR = 2·06–3·86.

A comorbidity was present in 59·4% of the participants (95% CI 49·4–68·7).

**Fig. 1.**
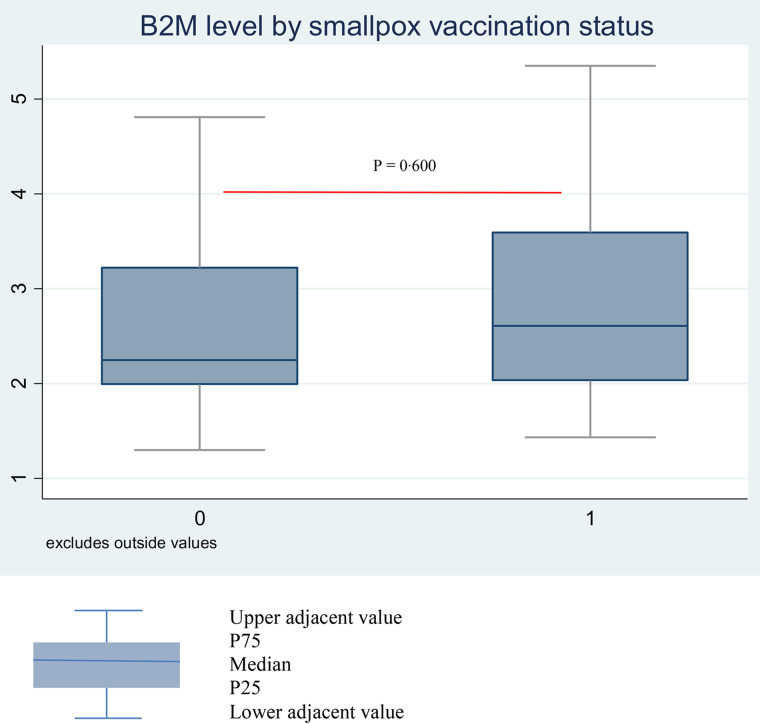
Median *β*2m level by smallpox scar status (absence *vs*. presence) among Senegalese ART-naïve PLHIV born before 1980.

Comparison of the characteristics of participants who presented with a smallpox vaccine scar and those who did not are summarised in Table 1. The former were older (median age: 48 *vs.* 42 years; *P* = 0·008) and more likely to have a BCG vaccine scar (71·2 *vs* 37·1; *P* = 0·001). They were more likely to present with a comorbidity (43·9% *vs.* 34·3%), with CD4 cell count <500, at advanced clinical stage (WHO clinical stage 3 or 4), with HIV-2 infection but less likely with a CD4/CD8 ratio <1; however, these differences were not statistically significant. Mean *β*2m levels were similar between the participants with scar: 3·3 (0·22) and without scar 3·2 (0·34); *P* = 0·985. The respective median levels were 2·6 mg/l (IQR = 2·1–3·9) and 2·3 (IQR = 2·0–3·8) (Fig. 1).

**Table 1.** Characteristics of the study population by the presence of smallpox vaccine scar

Characteristics	With scar		Without scar		*P* value
	*N*	Median (IQR*) or %	*N*	Median (IQR) or %	
Age (years)	66	48 (44–56)	35	42 (39–52)	0·018
35–45	22	33·3	22	62·9	
46–55	27	40·9	7	20·0	0·016
>55	17	25·8	6	17·1	
Sex	66		35		0·967
Male	28	42·4	15	42·9	
Female	38	57·6	20	57·1	
Education level	66		35		0·595
No education	16	24·2	8	22·9	
Elementary	25	37·9	10	28·6	
High school	19	28·8	11	31·4	
University	6	9·1	6	17·1	
Profession	66		35		0·799
No current occupation	24	36·4	10	28·6	
Informal work	33	50·0	18	51·4	
Other public or private sector employee	5	7·6	4	11·4	
Public or private sector executive	4	6·1	3	8·6	
IMC (kg/m^2^)	66	22·2 (19·3–27·4)	35	22·0 (19·8–26·9)	0·740
Comorbidities	66		35		0·347
Yes	29	43·9	12	34·3	
MDRD eGFR (ml/mn/1·73 m^2^)	66	89·2 (72·6–105·6)	35	85·0 (72·7–100·2)	0·881
<60	7	10·6	3	8·6	0·745
HIV serotype	66		35		0·361
HIV-1	47	71·2	28	80·0	
HIV-2	17	25·8	5	14·3	
HIV-1 + HIV-2	2	3·0	2	5·7	
WHO clinical stage	66		25		0·263
1 or 2	55	83·3	32	91·4	
3 or 4	11	16·7	3	8·6	
CD4 counts (cells/μl)	66	393·0 (138–580)	35	501·0 (184–568)	0·622
<500	40	60·6	16	48·5	0·251
CD4/CD8 ratio	66	0·42 (0·17–1·03)	33	0·43 (0·30–0·71)	0·795
<1	49	74·2	28	84·8	0·231
HIV VL (log copies/ml)	48	4·8 (3·5–5·8)	30	5·1 (4·3–5·9)	0·465
*β*2m level (mg/l)	66	2·6 (2·1–3·9)	35	2·3 (2·0–3·8)	0·600
BCG vaccine scar	66		35		0·001
Present	47	71·2	13	37·1	

* IQR: interquartile range.

### Association between smallpox vaccine scar and *β*2m level

We used different logistic regression models with different cut-off points to assess this association. The primary analysis used the median level as a cut-off point: the results of the association between the presence of smallpox vaccine scar and a *β*2m level ⩾2·59 mg/l are presented in Table 2. In univariable analysis, the crude OR for the association between the presence of smallpox vaccine scar and a *β*2m level ⩾2·59 mg/l was not statistically significant (OR 1·34; 95% CI 0·58–3·05; *P* = 0·485). A *β*2m level ⩾2·59 mg/l was however associated with the BMI and haemoglobin level: the corresponding OR by one unit increase were 0·92 (95% CI 0·86–0·99; *P* = 0·036) and 0·77 (95% CI 0·61–0·98; *P* = 0·031). It was also associated with plasma VL (the OR for one log copies/ml increase was 2·83; 95% CI 1·67–4·78; *P* < 0·001) and WHO clinical stage (the OR for stage ¾ *vs.* stage ½ = 4·31; 95% CI 1·12–16·53; *P* = 0·033).

**Table 2.** Logistic regression models evaluating the effect of smallpox scar on a β2m level ⩾2·59 mg/l among Senegalese ART-naïve PLHIV born before 1980

	Univariable analysis *N* = 101		Multivariable analysis* *N* = 99	
Smallpox vaccine scar	Crude OR (95% CI)	*P* value	Adjusted OR (95% CI)	*P* value
Absent	1		1	
Present	1·34 (0·58–3·05)	0·485	0·94 (0·32–2·77)	0·908

* Multivariate model adjusted for the following confounding variables (using a 10% change in estimate methods): age, sex, education level, WHO clinical stage, haemoglobin level, comorbidity and presence of BCG vaccine scar.

After adjustment, there was no association between a *β*2m level ⩾2·59 mg/l and the presence of smallpox vaccine scar: adjusted OR (aOR) 0·94; 95% CI 0·32–2·77 (Table 2).

The following variables did not modify this effect: age (*P* = 0·459), sex (*P* = 0·821) and the presence of BCG vaccine scar (*P* = 0·199); likelihood ratio test comparing the model with interactions and the model without interactions = 3·59; *P* = 0·310.

The exclusion of persons who were not at school age yet in 1980 from our population did not change our estimates with an aOR of 0·94 (95% CI 0·25–3·51; *P* = 0·931) (supplementary data). The aOR of the association among those with smallpox vaccine scar and BCG vaccine scar compared with those who did not have any scar was 0·62 (95% CI 0·16–2·36; *P* = 0·487) supplementary data. The aOR of the association among those with ⩾2 smallpox vaccine scars compared with those who did have ⩽1 scar was 0·59 (95% CI 0·17–2·06; *P* = 0·411) supplementary data. As shown in Table 3, when considering the fourth quintile as the cut-off point, the association between the presence of smallpox vaccine scar and a *β*2m level ⩾4·73 mg/l did not change substantially (aOR =0·66; 95% CI 0·10–4·74; *P* = 0·681). The results were also in accordance with the results of the linear regression model for the association between the presence of smallpox vaccine scar and *β*2m level treated as a continuous variable (Table 4).

**Table 3.** Logistic regression models evaluating the effect of smallpox scar on a β2m level *⩾*4·73 mg/l among Senegalese ART-naïve PLHIV born before 1980

	Univariable analysis *N* = 101		Multivariable analysis* *N* = 99	
Smallpox vaccine scar	Crude OR (95% CI)	*P* value	Adjusted OR (95% CI)	*P* value
Absent	1		1	
Present	0·75 (0·27–2·05)	0·575	0·66 (0·10–4·74)	0·681

* Multivariate model adjusted for the following confounding variables (using a 10% change in estimate methods): age, sex, BMI, education level, WHO clinical stage, haemoglobin level and presence of BCG vaccine scar.

**Table 4.** Linear regression models evaluating the effect of smallpox scar on a β2m level (continuous) among Senegalese ART-naïve PLHIV born before 1980

	Univariable analysis *N* = 101		Multivariable analysis* *N* = 99	
Smallpox vaccine scar	Crude *β* (95% CI)	*P* value	Adjusted *β* (95% CI)	*P* value
Absent	1		1	
Present	0·03 (−0·74 to 0·79)	0·945	−0·08 (−0·80 to 0·63)	0·818

* Multivariate model adjusted for the following confounding variables (using a 10% change in estimate methods): age, sex, BMI, marital status, occupation, education, HIV serotype, WHO clinical stage, haemoglobin level, comorbidity, plasma VL and presence of BCG vaccine scar.

## DISCUSSION

In this population of 101 ART-naïve PLHIV, there was no difference in the level of *β*2m between those who presented with a smallpox vaccine scar and those who did not. After adjustment, the presence of smallpox vaccine scar was not associated with the *β*2m level. Our study population was relatively homogeneous and two different clinicians independently assessed the presence of smallpox vaccine scar. The diameter of the biggest smallpox vaccine scar found in our study was concordant with previously published findings [6, 7, 19, 20]. We also collected information on occupation, education level and other comorbidities that might influence this association. The results of our analyses remained unchanged when considering people who were at least at school age when smallpox vaccination was stopped or when excluding people with BCG scar. Nor did they change when considering those with smallpox and BCG scars compared with those without any scar; nor when comparing those with <2 scars and those with ⩾2 smallpox vaccine scars. This limits potential bias due to misclassification of exposure as well as selection bias.

Several limitations affect this study: (1) we are attempting to establish an association between an exposure and an outcome measured more than 37 years after the exposure, (2) the sample size was low because it could demonstrate only an association with an OR⩾4 (two-side test, *α* = 0·05, *β* = 0·80, cut-off point = median level of *β*2m, 65·3% of the study population are exposed) while a sample size of 194 could demonstrate an OR⩾2·5 under the same conditions, and (3) there are no validated measures of smallpox vaccine scar resulting in potential classification errors. Such errors might be non-differential since the outcome variable (*β*2m level) was measured in an external laboratory without any information on the hypotheses studied. In this case, the effect would be underestimated. A temporal bias cannot be excluded: even if the first cases of AIDS were reported in the United States in 1981 (after smallpox vaccination was stopped), recent studies pointed out the origin of AIDS in the 1920s in Central Africa and a spread in Western Africa before 1980 [21, 22].

We did not find any studies that assessed the association between a previous smallpox vaccination and IA in the general population or in PLHIV. However, several studies examined the link between HIV infection and smallpox vaccination. Some studies reported that smallpox vaccination was associated with a positive effect on immune response and mortality in PLHIV. Durable and neutralising antibody titres after remote smallpox vaccination have been demonstrated in American PLHIV and would offer a certain protection against the disease [23]. Weinstein *et al.* found a slower *in vitro* replication of CCR5-tropic HIV-1 suggesting a certain protection against infection or disease progression [5].

Two clinical studies conducted in Guinea-Bissau reported long-term benefits of smallpox vaccination on mortality 26 years after the vaccination had occurred. They found that the presence of smallpox vaccine scar was associated with lower mortality in PLHIV. The first one took place in an urban setting and reported a mortality ratio of 0·60 in the global adult population comprising 502 PLHIV and the mortality ratio was not different in this group [7]. The second one reported a mortality ratio of 0·22 among 193 PLHIV in a rural area [6]. This was not found in a recent case-cohort study conducted on a subcohort of 5316 schoolchildren born in 1965–1976 and registered in the Copenhagen School Health Record Register (CSHRR): the adjusted mortality hazard ratio for smallpox vaccinated *vs.* not vaccinated was 0·75 (95% CI 0·47–1·22). This study did not confirm results from Guinea-Bissau, although the authors noted a trend to reduced mortality [24]. Another study in the same CSHRR reported that smallpox vaccination was associated with a reduced risk of infectious disease hospitalisation [25]. Both Danish studies used a more accurate measure of smallpox vaccination. Vaccination status was ascertained from vaccination cards where this information is systematically recorded. This is one strength of these studies compared with the studies from Guinea-Bissau which use scar as a measure of previous smallpox vaccination.

If previous smallpox vaccination reduces mortality in PLHIV, the most plausible mechanism would be by the reduction of IA which itself is the main driver of HIV infection. Our study did not find that smallpox vaccination was associated with decreased IA. It did not find the positive effects reported in previous studies [6, 7, 25]. The difference with our findings may be because immune stimulation consequent to smallpox vaccination has a limited duration that is not as long lasting as the period considered in our study. It has already been found that antiviral T-cell responses decline slowly while antiviral antibody responses remain stable for decades after smallpox vaccination or infection [26].

The differences in mortality previously observed might not be related to IA but to pathways unrelated to HIV disease progression. This is supported by the observation that the benefits observed in PLHIV are also observed in HIV-negative persons [6, 7, 24, 25]. Moreover, live vaccines such a smallpox vaccine and measles vaccine have long-term benefits on health even after the targeted infection is resolved, supporting the concept of non-targeted effect of vaccines [27]. In addition, smallpox vaccine scar was reported to confer a certain protection against other conditions not related to HIV [24, 25, 28–35].

Like every cross-sectional study, our study population was constituted by the persons who were alive at the moment of the study. Those with poorer health outcomes at the time of smallpox vaccination were more likely to be deceased before the measurement of the outcome. This may be the case for those to whom the smallpox vaccine was contraindicated.

Generally, the immunogenicity of vaccines is lower in PLHIV when viral replication is not controlled and when the CD4 lymphocyte count is <500 cells/*μ*l and even more when <200/μl. The protection obtained is of short duration and may require more frequent booster vaccinations than in immunocompetent persons [36]. An important proportion of our study population had ⩽500 CD4/μl. The lower immunogenicity of smallpox vaccine in PLHIV can be supported by the results of Kan *et al.* on US veterans who received two Dryvax vaccinations before 1991. Comparing smallpox neutralising antibodies between 20 PLHIV and 20 matched (on age and entry at military service) HIV negatives, neutralising antibodies were found in 95% of PLHIV *vs.* 100% of controls, and protective levels were found in 40% of PLHIV *vs.* 70% of controls [23]. These proportions would be probably lower in our population where 74·2% of participants with vaccine scars had only one scar.

Groups with a higher socio-economic level might have greater access to vaccination in general and would be expected to have better survival. This would help to explain why in our study population, participants with a smallpox vaccine scar were more likely to have a BCG vaccine scar. In our population, there was no difference in measures of socio-economic status (i.e. occupation and education) between the two groups and estimates were adjusted on these variables. However, these variables do not accurately reflect the socio-economic level at the moment of smallpox vaccination that had occurred decades before. This might impact measures of association in our study as well as in previous studies from Guinea-Bissau. Individuals who recover from smallpox should have immunologic profiles similar to those of vaccinated persons but will not present with smallpox scar [26, 37]. This might have a conservative effect on the association between smallpox vaccine scar and IA or mortality.

In this study, we hypothesised that ART-naïve PLHIV who had been vaccinated against smallpox had lower level of IA than those who had not. Using a cross-sectional study design, we did not find any association between the presence of smallpox scar and the *β*2m level. No previous study has analysed the association between IA level and smallpox vaccination, although studies have reported benefits of smallpox vaccinations on morbidity and mortality in different populations including PLHIV. These benefits are attributed to the non-targeted effects of smallpox vaccine whose underlying mechanisms are poorly understood. The results of our study suggest that the IA level might not be a significant determinant of the non-targeted effect of smallpox vaccination in PLHIV. However, more accurate measures of the history of smallpox vaccination and a better study design would permit to gain further insight into the relationship between smallpox vaccination and IA among PLHIV.

## Supplementary material

For supplementary material accompanying this paper visit https://doi.org/10.1017/S0950268817002795. click here to view supplementary material
