# Supplementary material for: Does smallpox vaccination modify HIV disease progression among ART-naive people living with HIV in Africa?
Source: Epidemiol Infect. 2017 Dec 13;146(2):218–26. doi: 10.1017/S0950268817002795 (PMC5791884; doi:10.1017/S0950268817002795)
Supplement: Supplementary file 1 [file S0950268817002795sup001.docx]

**Supplementary results**

Table 5 Logistic regression models evaluating the effect of smallpox scar on a β2m level ≥ 2.59 mg/L among Senegalese ART-naïve PLHIV born before 1974

|  | Univariable analysis  N = 79 | | Multivariable analysis*  N = 77 | |
| --- | --- | --- | --- | --- |
| Smallpox vaccine scar | Crude OR [95% CI] | p value | Adjusted OR [95% CI] | p value |
| Absent | 1 |  | 1 |  |
| Present | 0.99 [0.36 – 2.71] | 0.984 | 0.94 [0.25 – 3.51] | 0.931 |

*multivariate model adjusted for the following confounding variables (using a 10% change in estimate methods): age, sex, education level, WHO clinical stage, haemoglobin level, comorbidity and presence of BCG vaccine scar

Table 6 Logistic regression models evaluating the effect of smallpox vaccine scar and BCG vaccine scar on a β2m level ≥ 2.59 mg/L among Senegalese ART-naïve PLHIV born before 1980

|  | Univariable analysis  N = 69 | | Multivariable analysis*  N = 67 | |
| --- | --- | --- | --- | --- |
| Smallpox + BCG scars | Crude OR [95% CI] | p value | Adjusted OR [95% CI] | p value |
| Absent | 1 |  | 1 |  |
| Present | 0.79 [0.28 – 2.19] | 0.646 | 0.62 [0.16 – 2.36] | 0.487 |

*multivariate model adjusted for the following confounding variables (using a 10% change in estimate methods): age, sex, education level, WHO clinical stage, haemoglobin level, comorbidity and presence of BCG vaccine scar

Table 7 Logistic regression models evaluating the effect of multiple smallpox vaccine scars on a β2m level ≥ 2.59 mg/L among Senegalese ART-naïve PLHIV born before 1980

|  | Univariable analysis  N = 101 | | Multivariable analysis*  N = 99 | |
| --- | --- | --- | --- | --- |
| Smallpox vaccine scar | Crude OR [95% CI] | p value | Adjusted OR [95% CI] | p value |
| < 2 | 1 |  | 1 |  |
| ≥ 2 | 0.84 [0.30 – 2.41] | 0.756 | 0.59 [0.17 – 2.06] | 0.411 |

*multivariate model adjusted for the following confounding variables (using a 10% change in estimate methods): age, sex, education level, WHO clinical stage, haemoglobin level, comorbidity and presence of BCG vaccine scar
